# Fungal diversity and ecosystem function data from wine fermentation vats and microcosms

**DOI:** 10.1016/j.dib.2016.05.038

**Published:** 2016-05-24

**Authors:** Primrose J. Boynton, Duncan Greig

**Affiliations:** a Max Planck Institute for Evolutionary Biology, Plön 24306, Germany; b The Galton Laboratory, Department of Genetics, Evolution, and Environment, University College London, London WC1E 6BT, UK

## Abstract

Grape must is the precursor to wine, and consists of grape juice and its resident microbial community. We used Illumina MiSeq® to track changes in must fungal community composition over time in winery vats and laboratory microcosms. We also measured glucose consumption and biomass in microcosms derived directly from must, and glucose consumption in artificially assembled microcosms. Functional impacts of individual must yeasts in artificially assembled communities were calculated using a "keystone index," developed for “Species richness influences wine ecosystem function through a dominant species” [1]. Community composition data and functional measurements are included in this article. DNA sequences were deposited in GenBank (GenBank: SRP073276). Discussion of must succession and ecosystem functioning in must are provided in [1].

**Specifications Table**

**Table:** 

Subject area	*Biology*
More specific subject area	*Ecology, Microbiology, Mycology, Oenology*
Type of data	*Tables*
How data was acquired	*Illumina MiSeq*® *sequencing; colorimetric glucose (HK) assay kit; microbalance weighing; colony-forming unit (CFU) counting*
Data format	*Raw and analysed*
Experimental factors	*Fermentation age in winery vats; inoculum dilution in microcosms; added genus in artificially assembled communities*
Experimental features	*Fermenting grape must from winery vats and laboratory microcosms was sequenced for fungal-specific amplicons. Glucose and biomass were measured in microcosms. Glucose was also measured in artificially assembled communities derived from individual yeast isolates and microcosms.*
Data source location	*San Polino Winery, Montalcino, Italy*
Data accessibility	*Data are within this article. Raw sequence data are available at GenBank via the accession number GenBank: SRP073276.*

**Value of the data**

• This dataset is one of very few must fungal datasets measured over successional time using high-throughput sequencing.
• Fungal succession over time in fermenting must can be contrasted with datasets. from different winery environments or gathered using different enumeration techniques (*e.g.,* culture-dependent enumeration).
• Measured sugar utilization of yeasts and microbial communities can serve as a starting point for studies of yeast function during wine development.
• The keystone index can be used to compare disproportionate functional impacts among microbes from a variety of environments.

## 1 Data

Data include fungal DNA amplicon sequences, OTUs, and taxonomic data from fermenting must in a winery, in a laboratory microcosm experiment, and in control communities. Associated experimental and metadata are provided in separate tables. Experimental data include microcosm biomass and glucose concentrations, plus the data needed to calculate a "keystone index," described in [1]below, for twenty microcosm yeast isolates. Metadata include winery vat identity, fermentation age, microcosm treatment, microcosm age, and microcosm replicate.

## 2 Experimental design, materials and methods

### 2.1 Fermenting grape must

All must samples were collected in October and November, 2013 from the San Polino winery in Montalcino, Italy. All winery fermentation vats were filled with must from Sangiovese grapes harvested from five vineyards, all within 5 km of the winery. Vat volumes range from 3000 to 3800 l. We collected must samples from five vats approximately every 12–24 h over 13 days starting from the day the first vat was completely filled. One ml of grape must was collected at each timepoint. To prevent further fermentation during storage and transport, we centrifuged must samples for 5 min at 6000 rpm in a tabletop microcentrifuge and fixed the pelleted cells in 250–500 μl 100% ethanol. Samples were stored at ambient temperature until DNA extraction (19 days or less), and alcohol was removed from each sample before DNA extraction. DNA was extracted using the MasterPure™ Yeast DNA Purification Kit (Epicentre, Madison, Wisconsin, USA) following the manufacturer׳s instructions.

Must samples were also collected from six vats or vat mixtures once fermentation was completed, after the winemakers had filtered the fermented must. Post-filtration samples were transported at ambient temperature without treatment for seven days before DNA extraction. The winemakers combined the contents of some vats during filtration, and two post-filtration samples were mixtures of two vats each. We assigned each of these two mixtures to the vat which contributed the most volume to the mixture (*i.e.,* a sample consisting of 54% Vat 17 must and 46% Vat 1 must was analysed as Vat 17 and a sample consisting of 67% Vat 22 must and 33% Vat 20 must was analysed as Vat 22). The total number of must samples collected ranged from 6 to 23 per vat. Two additional vats were only sampled once, after filtration. A summary of all fermentation vat samples including fermentation age and vat identity is provided in Table 1.

### 2.2 Microcosm experiment

We sequenced fungal diversity and measured biomass and glucose consumption in small volumes of fermenting grape must (microcosms). We prepared ten replicates each of five dilution treatments plus uninoculated controls (Fig. S1 in [1]). Treatments included undilute unsterilized grape must and unsterilized must serially diluted 1:10, 1:10^3^, 1:10^5^, and 1:10^7^ with 0.22-μm-filter-sterilized must (D0, D1, D3, D5, and D7, respectively). One millilitre was removed from each inoculated microcosm for DNA sequencing before incubation, and the remaining 10 ml microcosms were incubated for 14 days at 30 °C with 200 rpm shaking. Inoculum sizes ranged from about 50 to 5×10^8^ colony-forming units (CFUs) per 10 ml microcosm. All must originated from a single vat (Vat 17). Must was collected 64 h after the vat was filled, and transported on ice for 24 h before microcosm preparation.

In addition to the cells harvested before incubation, we also harvested cells for DNA sequencing and measured microcosm biomasses and glucose concentrations after 14 days. Cells were harvested from all inoculated microcosms by centrifuging 1 ml of each microcosm (10 min at 16,837 rcf) and removing the supernatant. DNA was extracted as above. To measure biomass, we centrifuged a second 1 ml from each microcosm, dried each pellet at 80 °C for 38 h, and weighed pellets on a microbalance. We corrected biomass values by subtracting average uninoculated control values from each treated biomass value, but we ignored biomass data of D0 microcosms because these microcosms contained undilute grape solids. Supernatants were retained for glucose concentration assays. We decolourized supernatants by incubating 250 μl of filter-sterilized supernatant with 25–50 mg activated carbon pellets for 24 h. Glucose concentration was then measured using a Glucose (HK) Assay Kit (Sigma®, St. Louis, Missouri, USA), according to the manufacturer׳s instructions. Microcosm glucose values less than 0.14 mg/ml were assumed to be below the limit of kit detection, and were assigned a value of zero. We transformed microcosm glucose into percentage total glucose consumed by normalizing glucose concentrations to uninoculated controls. A summary of all sequenced microcosm samples is in Table 2, and glucose and biomass data are in Table 3.

### 2.3 Constructed control samples

Constructed control samples were known numbers of CFUs of three grape must yeasts (*S. cerevisiae*, *Hanseniaspora uvarum*, and *Metschnikowia sp.*) in grape must. DNA was extracted from constructed-control samples as described above for microcosm samples. CFU numbers are provided for each constructed control sample in Table 4.

### 2.4 MiSeq® amplicon sequencing, filtering, and OTU table production

Fungal ITS2 amplicons of 65 vat samples, 100 microcosm samples, and four constructed control samples were sequenced using MiSeq® (Illumina®, San Diego, California, USA). LGC Genomics (Berlin, Germany) prepared and sequenced a barcoded amplicon library consisting of all 169 samples amplified using the fungal-specific primer pair fITS7/ITS4 [2], [3]. Technicians at LGC Genomics diluted each DNA extract 1:50, and amplified samples using barcoded primers. Both forward and reverse barcodes were unique for each sample. PCR reactions consisted of 1 μl dilute template, 15 pmol each barcoded primer, 1.5 units MyTaq™ DNA polymerase (Bioline, London, UK), and 2 μl BioStab PCR Optimizer II (Sigma-Aldrich, St. Louis, Missouri, USA) in 20 μl MyTaq buffer. Reactions were cycled for 2 min at 96 °C, then for 40 cycles of 96 °C for 15 s, 50 °C for 30 s, and 70 °C for 60 s. Amplicon concentration was then determined using gel electrophoresis, and about 20 ng of each amplicon was pooled into 48-sample amplicon pools. Amplicon pools were purified using both AMPure® XP beads (Beckman-Coulter, Krefeld, Germany) and MinElute® columns (Qiagen, Hilden, Germany) to remove primer dimers. LGC then constructed Illumina libraries using the Ovation® Rapid DR Multiplex System (Qiagen, Hilden, Germany), and ran samples on Illumina MiSeq® cartridges using V2 or V3 chemistry.

Sequencing produced a total of 8,098,202 paired-end contigs. LGC genomics sorted fastq files by barcode, removed adapter and barcode sequences, and discarded sequences with missing or incompatible barcodes using BCL2Fastq Version 1.8.4 (Illumina, San Diego, California, USA) and in-house scripts. We then used Mothur version 1.33.3 to join paired ends into contigs [4]. Mothur also removed 757,652 sequences with ambiguous bases, homopolymers longer than 18 bp, or length not between 250 and 550 bases. The remaining sequence dataset was composed of 1,580,442 unique sequences. Of the unique sequences, we removed 19,617 sequences that were predicted to be chimeric using the *de novo* UCHIME interface in Mothur [5], leaving 1,560,825 unique and 7,318,146 total sequences. We clustered operational taxonomic units (OTUs) at 98.5% similarity using the blast-based reference method in QIIME [6], [7]. OTUs were clustered against the dynamic UNITE database version 6, release date September 10, 2014, containing 21,185 total reference and representative sequences [8]. OTUs were assigned the same taxonomic identity as the UNITE sequence to which they were clustered. Sequences below 98.5% similarity to a UNITE sequence were discarded (1,285,298 sequences). OTUs represented only once in our dataset (singleton OTUs) were assumed to be sequencing errors, and were removed (87 sequences). The final dataset was composed of 6,032,761 sequences clustered into 524 OTUs. The operational taxonomic unit (OTU) table including taxonomy assignments to species provided in Table 5, formatted as a biom file. Fastq files are available at GenBank via the accession number GenBank: SRP073276. Analyses and discussion of all sequencing data are in [1].

### 2.5 Keystone species assay

We also reinoculated individual microcosm yeast isolates into communities derived from four of the microcosms; each isolate was assigned a "keystone index" to quantify disproportionate influences of a yeast on community glucose consumption. We included five isolates from each of the genera *Saccharomyces*, *Hanseniaspora*, *Nakazawaea*, and *Cryptococcus* as experimental replicates within a genus. Isolates were cultured and identified in [1]. We combined yeast isoaltes with inocula derived from each of four microcosms (microcosm replicates 1, 2, 4, and 8). Inocula were prepared, and artificial communities were grown, in filter-sterilized commercial grape juice (Aldi-Nord, Essen, Germany). To produce inocula, each yeast isolate and 30 μl of each frozen microcosm stock was individually grown in grape juice overnight at room temperature. We determined yeast and microcosm inoculum sizes by diluting inocula and counting CFUs on YPD media (1% yeast extract, 2% peptone, 2% dextrose, 2.5% agar).

To produce artificial communities, small amounts of each yeast inoculum were mixed with larger amounts of each microcosm inoculum; we aimed to inoculate each artificial community with 10% yeast CFUs and 90% microcosm CFUs, although there was considerable variation in relative inoculum sizes (mean=11%, standard deviation=13%). A total of 50 artificial communities were produced (including four yeast genera, five replicate isolates per yeast, and four microcosm inocula. The experimental design was not fully factorial). We also produced control artificial communities composed of uninoculated juice, each yeast alone (four yeast genera×five replicate isolates), and each microcosm inoculum alone (four microcosm inocula×five identical replicates). Artificial communities were grown at 30 °C for seven days with 200 rpm shaking. Final glucose concentration was assayed as described above. Raw data are provided in Table 6, and keystone indices are provided in Table 7.

## Figures and Tables

**Fig. S1:**
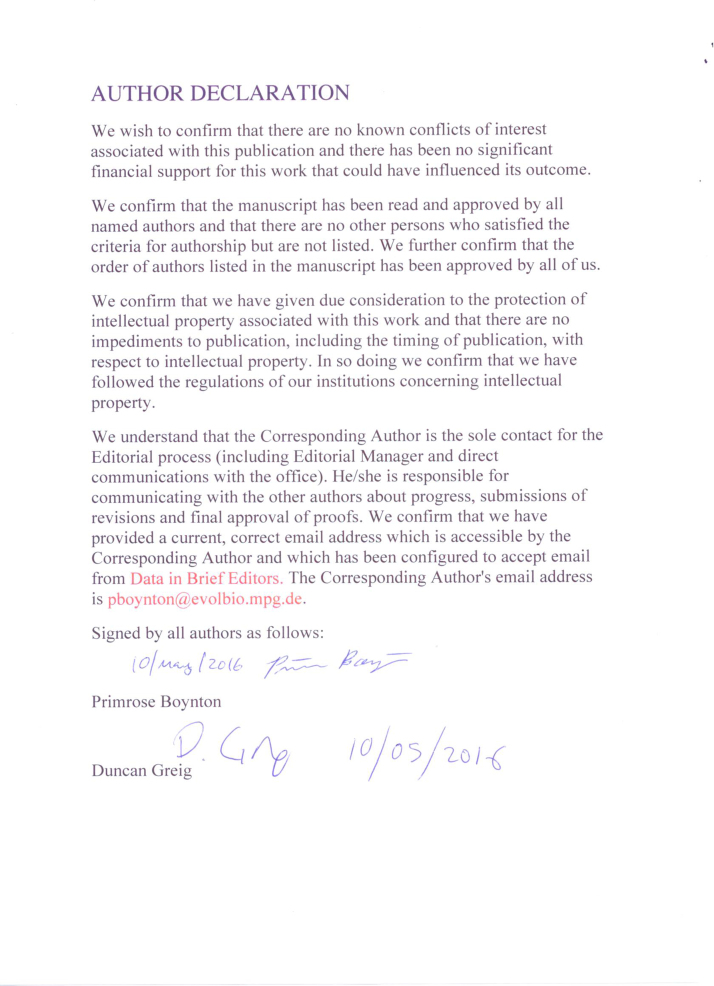


## Footnotes

Appendix A Supplementary material associated with this article can be found in the online version at doi:10.1016/j.dib.2016.05.038.
